# Development of an ELISA for the quantification of mycolactone, the cytotoxic macrolide toxin of *Mycobacterium ulcerans*

**DOI:** 10.1371/journal.pntd.0008357

**Published:** 2020-06-26

**Authors:** Louisa Warryn, Jean-Pierre Dangy, Philipp Gersbach, Matthias Gehringer, Anja Schäfer, Marie-Thérèse Ruf, Nicolas Ruggli, Karl-Heinz Altmann, Gerd Pluschke

**Affiliations:** 1 Swiss Tropical and Public Health Institute, Basel, Switzerland; 2 University of Basel, Basel, Switzerland; 3 Department of Chemistry and Applied Biosciences, Institute of Pharmaceutical Sciences, Swiss Federal Institute of Technology (ETH) Zürich, Zürich, Switzerland; 4 The Institute of Virology and Immunology IVI, Mittelhäusern, Switzerland; University of Melbourne, AUSTRALIA

## Abstract

Mycolactones, macrolide cytotoxins, are key virulence factors of *Mycobacterium ulcerans*, the etiological agent of the chronic necrotizing skin disease Buruli ulcer. There is urgent need for a simple point-of-care laboratory test for Buruli ulcer and mycolactone represents a promising target for the development of an immunological assay. However, for a long time, all efforts to generate mycolactone-specific antibodies have failed. By using a protein conjugate of a truncated non-toxic synthetic mycolactone derivative, we recently described generation of a set of mycolactone-specific monoclonal antibodies. Using the first mycolactone-specific monoclonal antibodies that we have described before, we were able to develop an antigen competition assay that detects mycolactones. By the systematic selection of a capturing antibody and a reporter molecule, and the optimization of assay conditions, we developed an ELISA that detects common natural variants of mycolactone with a limit of detection in the low nanomolar range. The mycolactone-specific ELISA described here will be a very useful tool for research on the biology of this macrolide toxin. After conversion into a simple point-of-care test format, the competition assay may have great potential as laboratory assay for both the diagnosis of Buruli ulcer and for the monitoring of treatment efficacy.

## Author summary

The macrolide toxin mycolactone is the key virulence factor of *Mycobacterium ulcerans*, the causative agent of the chronic necrotizing skin disease Buruli ulcer. Mycolactone has cytotoxic activity and is secreted by the mycobacteria, causing tissue necrosis and immunosuppression. For research on Buruli ulcer, there is urgent need for a simple tool to quantify mycolactone. Using the first mycolactone-specific monoclonal antibodies that we have described previously, we have optimized here an antigen competition ELISA that detects common natural variants of mycolactone at a low nanomolar scale. Sensitivity of the assay is sufficient to detect the toxin in *M*. *ulcerans* culture supernatants and in the tissue of experimentally infected animals. Converted into a simple point-of-care test format, the competition assay may in future also be suitable as a diagnostic laboratory test for Buruli ulcer.

## Introduction

*Mycobacterium ulcerans* is the etiological agent of the chronic necrotizing skin disease Buruli ulcer (BU) that primarily affects children in West and Central Africa [1]. Genomic analyses have shown that *M*. *ulcerans* has emerged from a common ancestor with the fish pathogen *Mycobacterium marinum* [2, 3] by acquisition of a virulence plasmid carrying genes that encode polyketide-modifying enzymes and the giant polyketide synthases responsible for the synthesis of the lipid toxin mycolactone [4]. While *M*. *marinum* occasionally causes limited granulomatous skin lesions in humans [5], chronic *M*. *ulcerans* infections are associated with a much more severe pathology. Mycolactone plays a key role in the chronic necrotizing pathogenesis of BU and, in addition, analgesic and immunosuppressive effects are attributed to the toxin [6]. There is evidence of multiple modes of action of mycolactone, including inhibition of Sec61-mediated protein translocation, uncontrolled assembly of actin by binding to the Wiskott-Aldrich syndrome protein (WASP) family, and induction of apoptosis through increased expression of the pro-apoptotic regulator Bim [6, 7]. Mycolactone is an amphiphilic molecule, prone to forming aggregates in aqueous solutions [8, 9], to binding to soluble proteins [10], and to inserting into lipid bilayers [8, 11]. At an air/buffer interface, mycolactone has been shown to have surfactant properties with an apparent surface saturation concentration of 1 μM [8].

Early case detection and rapid initiation of antibiotic treatment are currently the key elements of BU control. The disease presents in a variety of clinical manifestations, complicating the clinical diagnosis [12]. Laboratory tests routinely used for confirmation of clinical diagnosis include primarily the microscopic detection of acid-fast bacilli (AFB) and *M*. *ulcerans*-specific PCR tests. While microscopy–the only diagnostic test that can currently be performed routinely at hospital level–has limited sensitivity, PCR detecting the insertion sequence IS*2404* is highly sensitive and specific [13]. However, PCR requires sophisticated laboratory infrastructure and well-trained personnel and is not reliable without strict quality control [14]. In resource-poor BU endemic countries, the test is only available at a few reference centres, which poses major logistical problems. Therefore, there is urgent need for a simple and rapid diagnostic test for BU that can be performed at local hospital level or in the field [13]. Mycolactone represents an ideal target for such an assay, since it seems to be unique to *M*. *ulcerans*. A mycolactone-specific assay may also be highly suitable for monitoring treatment efficacy and to diagnose relapses, since mycolactone levels in the affected tissue decline during successful specific therapy [15, 16].

Mycolactones consist of a core structure, a short C-linked upper side chain, and a longer C5-O-linked lower acyl side chain. Geographical lineages of *M*. *ulcerans* produce different pools of molecular variants of mycolactone, which differ in the structure of the lower polyunsaturated side chain but are otherwise structurally conserved [17]. For the generation of mycolactone-specific monoclonal antibodies (mAbs), we have used a truncated synthetic mycolactone in which the lower polymorphic side chain was replaced by a linker molecule [18]. Therefore, the epitopes recognized by these mAbs appear to comprise determinants of the conserved core and upper side chain. Consequently, common natural molecular species of mycolactone were recognized [18]. Here, our aim was to use the mAbs as antigen capture reagents to develop an immunological assay for the quantification of mycolactones.

## Materials and methods

### Ethical statement

Animal experiments performed were approved by the animal welfare committees of the Canton of Basel (authorization number 2375) and the Canton of Bern (authorization number BE95/17). They were conducted in compliance with the Swiss Animal Welfare Act (TSchG), Animal Welfare Ordinance (TSchV), and the Animal Experimentation Ordinance (TVV).

### Preparation of mycolactone stock solutions

**Fig 1:**
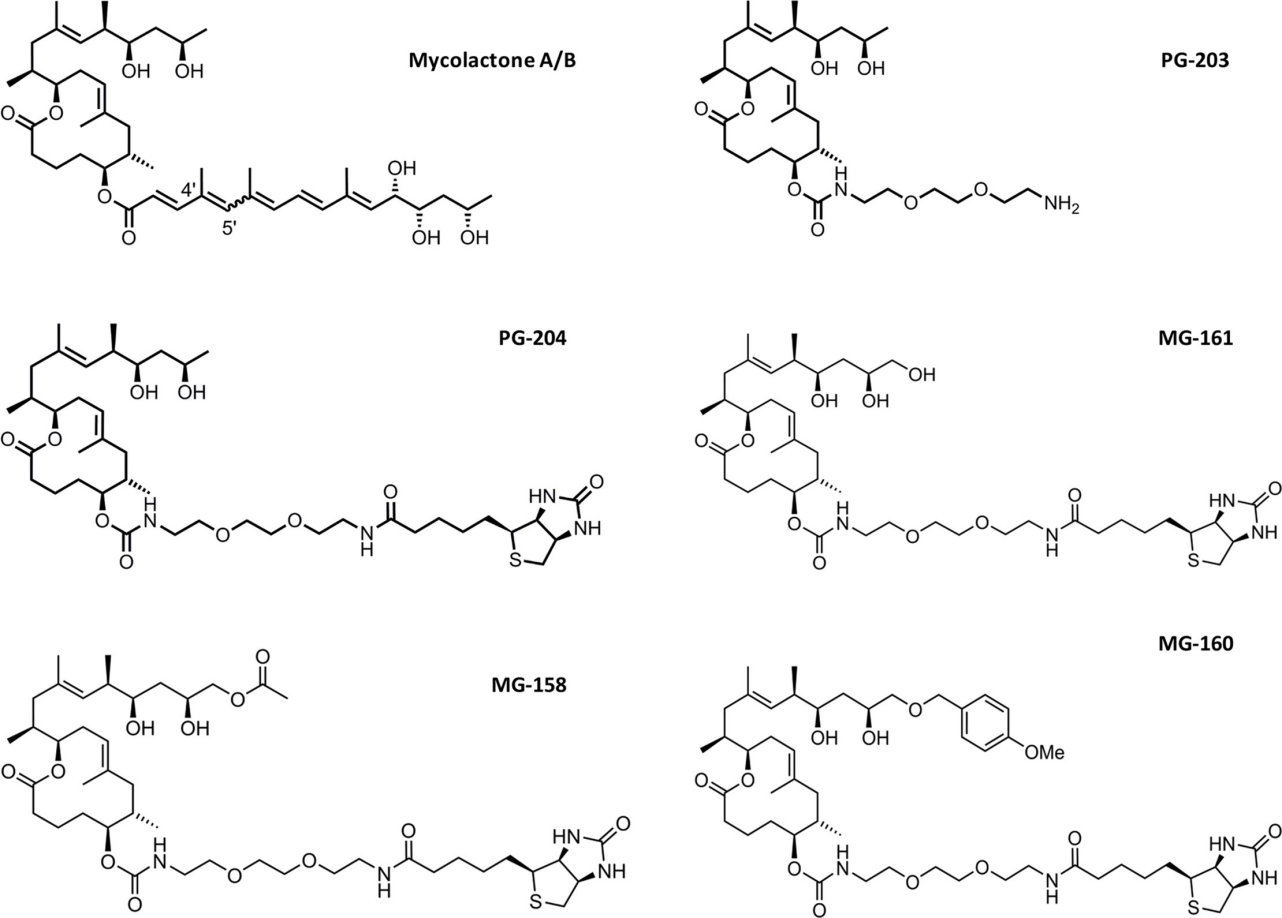
Structures of mycolactone A/B and of synthetic derivatives.

Production of synthetic mycolactones and mycolactone derivatives (Fig 1) has been described elsewhere [7, 17, 19, 20]. All compounds were HPLC-purified.

### Monoclonal antibodies

Generation of the 12 mouse mAbs designated JD5.1 to JD5.12 has been described previously [18]. MAbs were purified from hybridoma culture supernatants by affinity chromatography using a HiTrap Protein A HP column (GE Healthcare).

### Initial competition ELISA

MaxiSorp immunoassay plates (Thermo Scientific) were coated with 100 μL mAb (10 μg/ml) overnight at 4°C. After washing the plate twice with PBS-0.05% Tween-20 (PBST), the wells were blocked with SuperBlock T20 (TBS) blocking buffer (Thermo Scientific) for 1 h at 37°C. After another washing step, serial dilutions of the sample were made in PBST and added to the plate (100 μL/well), and then incubated in the dark for 2 h at 37°C. Without washing, 100 μL/well of a 200 pg/ml solution of the reporter molecule PG-204 (Fig 1) was added to the plate and incubated for an additional 30 min. Subsequently, plates were washed four times, and bound PG-204 was detected using alkaline phosphatase-coupled streptavidin (SouthernBiotech)/para-nitrophenylphosphate (pNPP) detection after 1 h incubation at 37°C.

### Optimized competition ELISA

For assay optimization phosphate-, tris(hydroxymethyl)aminomethan (TRIS)- and triethanolamine (TEA)-based buffers with different concentrations of dimethyl sulfoxide (DMSO) were compared. Furthermore, different reporter molecules, reagent concentrations, detection systems, and the use of detergents (Tween-20, Triton X-100, Triton X-114, IGEPAL, Brij 35, CHAPSO; Sigma-Aldrich) were evaluated. Following these multiple optimization steps, a new standard protocol was defined: MaxiSorp immunoassay plates were coated with 5 μg/ml of mAb JD5.1 overnight at 4°C. After washing the plate twice with PBST, the plate was blocked with SuperBlock T20 (TBS) blocking buffer (Thermo Scientific) for 1 h at 37°C. After another washing step, serial dilutions of the samples in LW buffer (0.2 M TEA, pH 7.5, with 20% DMSO) were added to the plates and incubated in the dark for 2 h at 37°C. Without washing, 100 μL of an 80 ng/ml solution of the reporter molecule MG-161 (Fig 1) in LW buffer was added to the plate and incubated for an additional 45 min. Subsequently, plates were washed four times, and bound MG-161 was detected after 1 h incubation at 37°C by horseradish peroxidase-coupled streptavidin (SouthernBiotech) diluted 1:5000 in PBST. Signal development was done with 3,3’,5,5’-tetramethylbenzidine (TMB) for 5 min after which the reaction was stopped with 0.5 M sulphuric acid.

### Binding of mAbs to reporter molecules in ELISA

NeutrAvidin-coated plates (Thermo Scientific) were coated with 1 μg/ml of the different biotinylated mycolactone derivatives–MG-158, MG-160, and MG-161 (Fig 1). Each of the 12 mAbs was serially diluted in PBST, added to the coated plates, and incubated for 2 h. After a washing step, bound mAbs were detected using horseradish peroxidase-conjugated goat anti-mouse IgG antibodies (SouthernBiotech) after 1 h incubation at 37°C. Signal development was done with TMB for 7 min after which the reaction was stopped with 0.5 M sulfuric acid.

### Competition ELISA using mycolactone-coated plates

NeutrAvidin-coated plates (Thermo Scientific) were coated with 1 μg/ml of the biotinylated mycolactone derivative MG-161 (Fig 1). A dilution series of mycolactone was prepared to which the mAb JD5.1 was added at a fixed concentration, and both were incubated together at 37°C for 2 hours to allow for binding. Subsequently, the mixture was transferred to the MG-161-coated plates and incubated for 1 hour at 37°C. Bound mAbs were detected using a secondary antibody conjugated with horseradish peroxidase (HRP), and detection was done with TMB.

### Cultivation of *M*. *ulcerans* and analysis of secreted mycolactone

An African *M*. *ulcerans* strain (S1013) and an Australian strain (S1251) were grown at 30°C in BACTEC liquid culture media (Becton Dickinson). Both strains have only been minimally passaged after isolation from BU lesions. For the detection of mycolactones in the bacterial culture supernatants, 500 μl of well-grown *M*. *ulcerans* cultures were centrifuged at 13,300 x *g* to pellet the bacteria, and the supernatant was filtered using sterile 0.22 μm syringe filters. The sterile-filtered supernatants were used directly in the assay.

### Analysis of *M*. *ulcerans* in infected mouse footpads

BALB/c mice, age 7–8 weeks (Janvier Labs, Le Genest-Saint-Isle, France) were inoculated in the left hind footpad with about 5 x 10^3^ CFU of the *M*. *ulcerans* isolate S1013 from Cameroon. The progression of footpad swelling was tracked for 13 weeks (S1 Fig), when mice were euthanized, and the footpads were collected and stored in absolute ethanol for 9 weeks. Uninfected right hind footpads served as controls. Subsequently, the ethanol was recovered and concentrated by vacuum centrifugation (SpeedVac, Thermo Scientific). Footpads were minced and homogenized in a bead-beater (Precellys MK-28R) in 1.5 ml of ethyl acetate. The supernatant was filtered and concentrated by vacuum centrifugation. Each extract was re-suspended in 60 μl of DMSO, and half this volume was used in the assay. For the quantification of the concentration of mycolactone detected in the extracts, standard curves were generated with synthetic mycolactone A/B and used to derive a regression equation for the determination of the concentration in the extracts.

## Results

We have tested a panel of 12 anti-mycolactone mAbs (JD5.1 –JD5.12; Table 1) described previously [18] for suitability as capturing reagent for a mycolactone-specific competition assay. The mAbs were generated by immunization of mice with PG-203, a truncated and non-cytotoxic mycolactone derivative coupled to BSA via a linker replacing the C5-O-linked polyunsaturated acyl side chain (Fig 1), and by ELISA screening of hybridomas using PG-204 (Fig 1), which is a biotinylated derivative of PG-203 [18]. As predicted from the structure of PG-203 and PG-204, the presence/absence or detailed structure of the C5-O-linked acyl side chain does not affect binding of the mAbs and, since the core and upper side chain of mycolactone are completely conserved, all tested mycolactone variants showed binding [18]. While a competition ELISA format with the mAbs was initially only used to assess their fine specificity [18], we describe here the optimization of this assay format for the detection and quantification of mycolactones in biological samples.

**Table 1:** Binding of the anti-mycolactone mAbs to different reporter molecules. NeutrAvidin-coated plates were coated with the different reporter molecules at 1 μg/ml and 3-fold dilution series of the different mAbs starting from 5 μg/ml were added and allowed to bind. Bound mAbs were detected with an HRP-conjugated secondary antibody and TMB. Binding was graded based on the absorbance (OD) values measured at 450 nm as follows: (-) OD < 0.5, (+) OD 0.5–1, (++) OD >1–1.5, (+++) OD > 1.5.

	PG-204	MG-161	MG-158	MG-160
JD5.1	+++	+++	+++	-
JD5.2	+++	+++	-	-
JD5.3	+++	+++	+	-
JD5.4	+++	+++	++	-
JD5.5	+++	+++	++	-
JD5.6	+++	+++	+	-
JD5.7	+++	+++	-	-
JD5.8	+++	+++	++	-
JD5.9	+++	+++	+++	-
JD5.10	+++	+++	+++	-
JD5.11	+++	+++	+++	+
JD5.12	+++	+++	+	-

### Optimization of the reporter molecules

**Fig 2:**
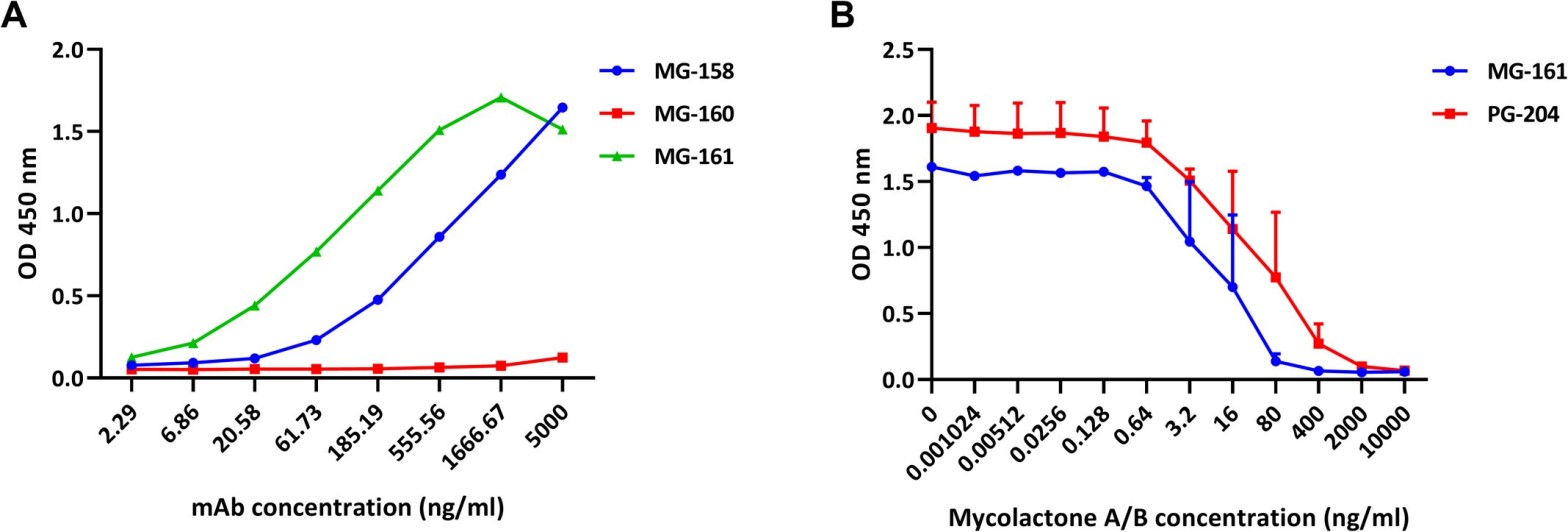
Binding properties of mAb JD5.1. (A) Binding of mAb JD5.1 to the different reporter molecules. NeutrAvidin-coated plates were coated with the different reporter molecules (1 μg/ml), a serial dilution of mAb JD5.1 was allowed to bind, and bound mAb was detected with an HRP-conjugated secondary antibody. (B) Sensitivity of the JD5.1-based competition assay with PG-204 (100 pg/ml) or MG-161 (40 ng/ml) as reporter molecules. JD5.1 bound to MaxiSorp plates was allowed to react with dilution series of mycolactone A/B for 2 hours after which PG-204 or MG-161 was added for 45 min; bound reporter was detected with HRP-conjugated streptavidin and TMB. Experiments were done in duplicate (two separate runs on different days) and the results shown are the mean of both runs, with error bars indicating the range.

To find a potentially more suitable substitute for the initially used reporter molecule PG-204 (Fig 1) for the competition assay, we screened the panel of 12 mAbs for recognition of related derivatives with modifications at the C20 position of the upper side chain (MG-158, MG-160, and MG-161; Fig 1). While all mAbs showed strong binding to MG-161, which carries an additional hydroxyl group, only one mAb showed limited binding to MG-160 carrying a bulky *p*-methoxybenzyl ether group. A wide variation in binding was observed with the different mAbs for MG-158, carrying an additional acetoxy group (Table 1). Overall, competition assays with mAb JD5.1, which bound to both MG-158 and MG-161 (Fig 2A) showed the highest sensitivity. This mAb appears to have a higher affinity for PG-204 than for mycolactone, which explains why as little as 100 pg/ml of PG-204 can out-compete a large excess of mycolactone for binding to the mAb [18]. We surmised that using a less tightly bound reporter molecule would allow for lower concentrations of mycolactone to be detected in the assay. In fact, MG-161 was found to be the best substitute for PG-204, resulting in an approximately 5-fold increase in the assay sensitivity (Fig 2B). However, to obtain optimal sensitivity, a 400-fold higher concentration of MG-161 than of PG-204 (40 ng/ml versus 100 pg/ml) was required.

### Optimization of the ELISA conditions

**Fig 3:**
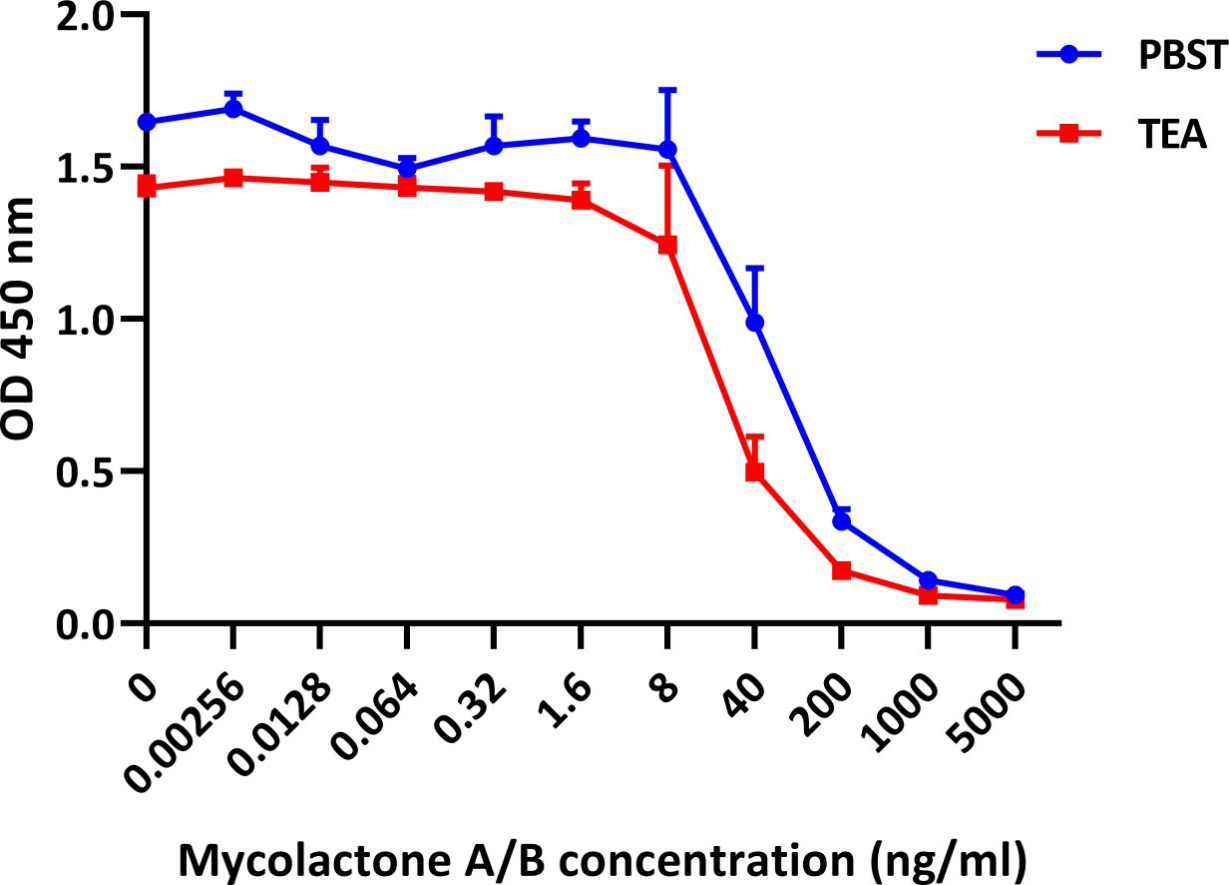
Improved assay sensitivity upon buffer optimization. Compared is the performance of the ELISA with the TEA-based LW buffer (0.2 M TEA with 20% DMSO) or with a PBST-based buffer (PBST with 20% DMSO). JD5.1 bound to MaxiSorp plates (coating concentration 5 μg/ml) was allowed to react with dilution series of mycolactone A/B in PBST buffer or TEA-based buffer for 2 hours after which MG-161 (40 ng/ml) was added for 45 min; bound reporter was detected with HRP-conjugated streptavidin and TMB. Results shown are the mean of duplicate experiments and error bars indicate the range.

We attempted to improve sensitivity of the competition assay further by reducing aggregation of mycolactone without denaturing the mAbs used in the ELISA. While ethanol, acetonitrile, and sodium dodecylsulphate improve the dispersion of the toxin in water [9], these solvents were, even in small concentrations, detrimental for the binding of the mAbs to mycolactone. Screening of a panel of non-denaturing detergents (Tween-20, Triton X-100, Triton X-114, IGEPAL, Brij 35, CHAPSO) at various concentrations showed that no other detergent or concentration out-performed 0.05%-Tween-20 previously selected for the PBS-based assay buffer [18]. Further systematic comparisons of phosphate-, TRIS-, and TEA-based buffers with different concentrations of dimethyl sulfoxide (DMSO) and Tween-20 resulted in the definition of the LW buffer, giving the highest assay sensitivity in the low nanogram scale (Fig 3). The buffer contains 20% DMSO and 0.2 M TEA, but no detergent. TEA is commonly used in emulsifiers to dissolve compounds that are poorly soluble in water. The sensitivity of the competition assay is only slightly reduced in the presence of serum (S2 Fig).

**Fig 4:**
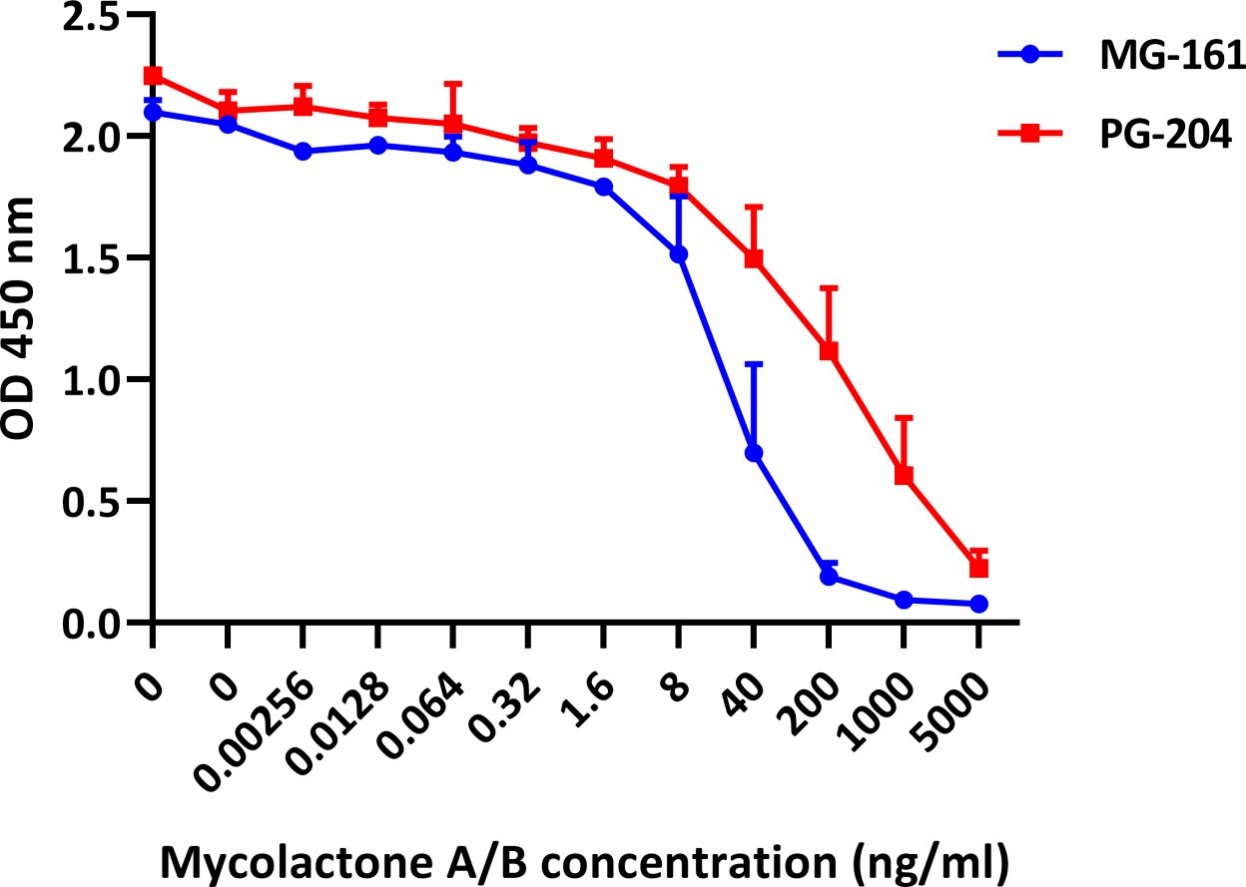
Indirect competition ELISA format. Serial dilutions of mycolactone were mixed with fixed concentrations of mAb JD5.1 and allowed to react for 2 h at 37°C after which the mix was transferred to NeutrAvidin plates coated with the target molecules MG-161 or PG-204 at 1 μg/ml. MAb bound to the plate was detected with an HRP-conjugated secondary antibody and TMB. Results shown are the mean of duplicate experiments and error bars indicate the range.

Small additional improvements of the assay sensitivity were achieved by switching from the alkaline phosphatase-based to the peroxidase-based antibody detection system, and by reducing the JD5.1 mAb coating concentration from 10 μg/ml to 5 μg/ml. A reversed competition format using biotinylated mycolactone bound to NeutrAvidin plates showed a sensitivity comparable to that with antibody-coated plates (Fig 4).

### Detection of mycolactone in biological samples

**Fig 5:**
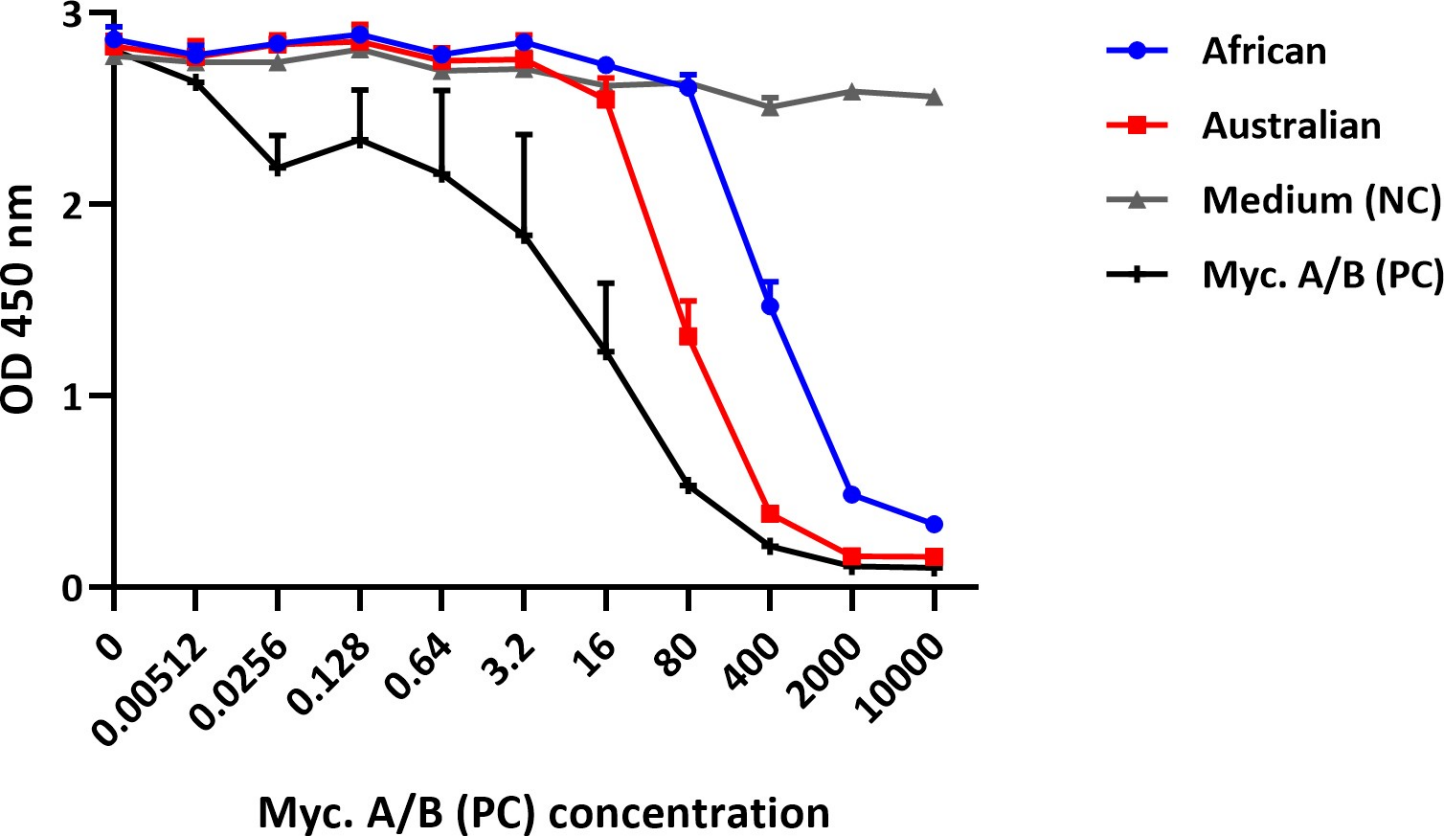
Detection of mycolactone in culture supernatants of *M*. *ulcerans* strains. Dilution series of culture supernatants of an African and an Australian *M*. *ulcerans* isolate, synthetic mycolactone A/B as positive control (PC), or plain medium as negative control (NC) were made in LW buffer and allowed to react with mAb JD5.1 bound to MaxiSorp plates for 2 hours after which MG-161 (40 ng/ml) was added for 45 min; bound reporter was detected with HRP-conjugated streptavidin and TMB. Results shown are the mean of duplicate experiments and error bars indicate the range.

Sterile-filtered culture supernatants of an African and an Australian *M*. *ulcerans* strain both gave positive readouts in the assay (Fig 5), demonstrating that both mycolactone A/B predominantly produced by African strains and mycolactone C predominantly produced by Australian strains that are together responsible for >95% of reported BU ulcer cases [1] are recognized. These supernatants were used directly in the assay without any prior lipid extraction.

**Fig 6:**
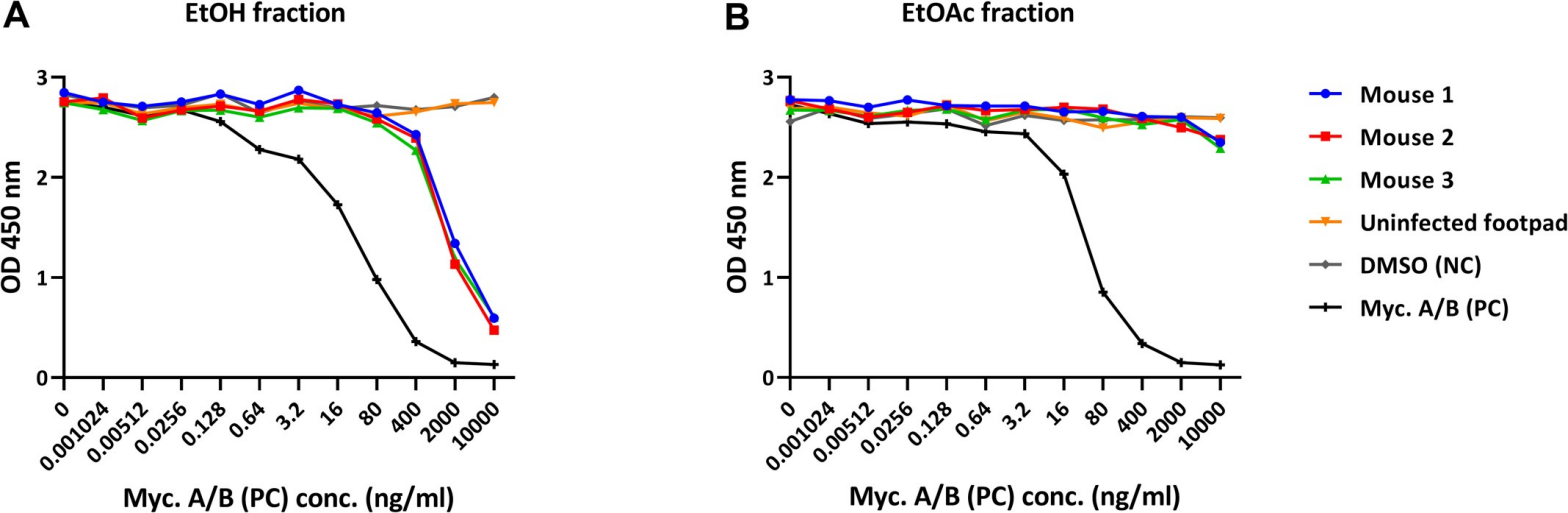
Detection of mycolactone in extracts from experimentally infected mouse footpads. Extracts were prepared by vacuum-centrifugation of the ethanol in which the footpads were stored (Fig 6A; EtOH fraction), or of the ethyl acetate in which the footpads were homogenized (Fig 6B; EtOAc fraction). Dilution series of the extracts or synthetic mycolactone A/B were made in LW buffer and allowed to react with mAb JD5.1 bound to MaxiSorp plates for 2 hours after which MG-161 (40 ng/ml) was added for 45 min; bound reporter was detected with HRP-conjugated streptavidin and TMB.

Mycolactone could also be specifically detected in extracts from mouse footpads infected with *M*. *ulcerans*. After extended storage (9 weeks) of the infected footpads in ethanol (EtOH), most of the mycolactone was found in the ethanol extract (Fig 6A), and only traces had remained in the footpad tissue, which was extracted with ethyl acetate (EtOAc) after homogenization of the footpads (Fig 6B). No mycolactone was detected in the extracts from uninfected control footpads (Fig 6). Using a standard curve generated with synthetic mycolactone A/B, the amount of mycolactone in the extracts was calculated (Table 2).

**Table 2:** Quantification of mycolactone in infected and uninfected mouse footpads.

Sample	EtOH fraction (ng)	EtOAc fraction (ng)
Mouse 1 footpad	55.9	1.0
Mouse 2 footpad	82.8	0.9
Mouse 3 footpad	53.5	1.4
Uninfected footpad	0	0

## Discussion

BU control measures rely mainly on early diagnosis of cases, preferably in the WHO Category I and II stages, and the prompt initiation of treatment. The current gold standard diagnostic test is the highly specific and sensitive PCR assay based on the detection of the high copy number insertion sequence IS*2404*. The main drawback in implementing this assay for routine diagnosis is the indispensability of well-equipped laboratories, skilled and experienced personnel, and rigid adherence to quality control measures. As a result, efforts have been made to develop BU diagnostics that would be low-cost and require simple instrumentation, while still giving results in a short time. Immunodiagnostic assays such as ELISA, which depend on the interactions between antibodies and their corresponding antigens, are viable alternatives to genetic test systems and are comparatively simpler to perform even at the primary healthcare or field levels. For BU, mycolactone makes an ideal antigen for specific immunodiagnosis given its uniqueness to the mycolactone-producing mycobacteria (MPM). Also, since the levels of mycolactone in tissues are reported to decline during specific treatment [15, 16], an assay quantifying mycolactone in BU lesions would be useful in monitoring treatment efficacy.

However, efforts to generate antibodies against this small, lipid-like, cytotoxic and immunosuppressive polyketide have long been unsuccessful. Using a novel approach which involved the use of a modified non-toxic variant of mycolactone coupled to a protein carrier to immunize mice, we have recently described for the first time the generation of mAbs against mycolactone [18]. Here, we describe the optimization of a competition ELISA based on these mAbs for the detection and quantification of mycolactone in biological samples. We sought to systematically optimize each step of the multi-step assay procedure, leading to modest gains per step but an overall 30-fold gain in sensitivity, allowing for the detection of nanogram levels of mycolactone in biological samples.

One key difficulty in detecting mycolactone by ELISA is its inherent hydrophobicity, largely due to the lower acyl side chain. ELISA, by default, is designed for hydrophilic molecules, typically proteins. Special methods have to be devised for the detection of hydrophobic molecules, such as the chloroform-ethanol coating method described for lipids [21, 22, 23]. However, we were aiming for more facile methodologies such that the resultant assay protocol would be relatively straightforward for the development of rapid diagnostic tests that can be performed in peripheral settings. As an amphiphilic molecule, mycolactone is thought to form micelle-like structures in aqueous solutions, with the acyl side chain sequestered within the core of these structures [9]. We attempted to disperse these structures using a variety of methods with the main challenge to find a solvent that would lead to improved mAb binding to the mycolactone molecules without denaturing the mAbs used in the ELISA. While detergents are routinely used to disperse lipid aggregates, none of the non-protein-denaturing detergents we tested led to an improvement in the sensitivity of our assay. In contrast, using 0.2 M TEA as the ELISA running buffer improved assay sensitivity compared to phosphate- and TRIS-based ELISA buffers. TEA is widely used as a buffering agent with surfactant properties in consumer products [24, 25] and in biomedical research [25, 26, 27, 28].

Switching the reporter molecule from PG-204 to MG-161, which is less tightly bound by the capturing mAb, also improved the overall assay sensitivity. Here, a drawback is that a 400-fold higher concentration of the synthetic mycolactone derivative is needed when switching to MG-161.

Conformational changes associated with the binding of mAbs to a plastic surface can lead to drastic changes in their antigen binding properties [29]. Therefore, we also explored the possibility of rearranging the assay set-up to allow the capturing mAb and mycolactone to react in solution, rather than at a solid-liquid interface with immobilized mAb. However, we found that this indirect competition format had no added benefits, and the need to use NeutrAvidin-coated plates for the coating with a biotinylated mycolactone derivative for mAb capturing increases the overall costs of the assay.

The utility of the improved competition ELISA as a research tool was assessed by measuring mycolactone concentrations directly in *M*. *ulcerans* culture supernatants and in footpads of experimentally infected mice. The amounts found in the infected footpads (50 to 100 ng/footpad) are comparable to those (49 ng/footpad) that were measured with thin-layer chromatography at a slightly earlier time point after infection [30]. The mycolactone ELISA thus is, in its optimized format, a valuable research tool that will allow to quantify mycolactone in large series of biological specimens. The assay may also be suitable as a diagnostic test for BU at the district hospital level. Reagents and assay conditions developed here may also be instrumental for the development of a simple point-of-care diagnostic test format, such as a lateral flow assay. While basic laboratory equipment and technical expertise is required to perform an ELISA, a simple lateral flow assay could be performed directly with wound exudate obtained by swabbing of ulcerative BU lesions or fine needle aspiration from closed lesions. While it has been shown, that mycolactone is extracted from serum samples with low efficacy [31], the sensitivity of the competition assay is only slightly reduced in the presence of serum. Our preliminary results indicate that no extraction with organic solvents may be required to perform these immunological tests.

In summary, the diagnosis of BU is still problematic, and development of BU diagnostics has not kept pace with the implementation of antibiotic treatments. Here, we describe a simple immunoassay for the specific and sensitive detection of mycolactone in biological samples. The generation of the first-ever described mAbs specific for mycolactone and chemical synthesis of mycolactone derivatives suitable as reporter molecules led to the development of a competition ELISA that we have systematically optimized here. While representing a valuable research tool for high-throughput quantification of mycolactone, this ELISA may also have potential as diagnostic assay for BU at district hospital level. Furthermore, the developed reagents and protocols may also enable development of a simple point-of-care test by converting the ELISA format into a lateral flow assay.

## Supporting information

S1 Fig Time course of footpad swelling in *M. ulcerans* infected mice. (TIF)

S2 Fig Sensitivity of the assay in the presence or absence of fetal bovine serum. Dilution series of synthetic mycolactone A/B were done in LW buffer alone or LW buffer containing 50% fetal bovine serum (FBS) were allowed to react with mAb JD5.1 bound to MaxiSorp plates for 2 hours; plain FBS was included as negative control. Subsequently, MG-161 (40 ng/ml) was added for 45 min, and bound reporter was detected with HRP-conjugated streptavidin and TMB.
(TIF)
