# Supplementary figures and images for: Development of an ELISA for the quantification of mycolactone, the cytotoxic macrolide toxin of Mycobacterium ulcerans
Source: PLoS Negl Trop Dis. 2020 Jun 26;14(6):e0008357. doi: 10.1371/journal.pntd.0008357 (PMC7347236; doi:10.1371/journal.pntd.0008357)

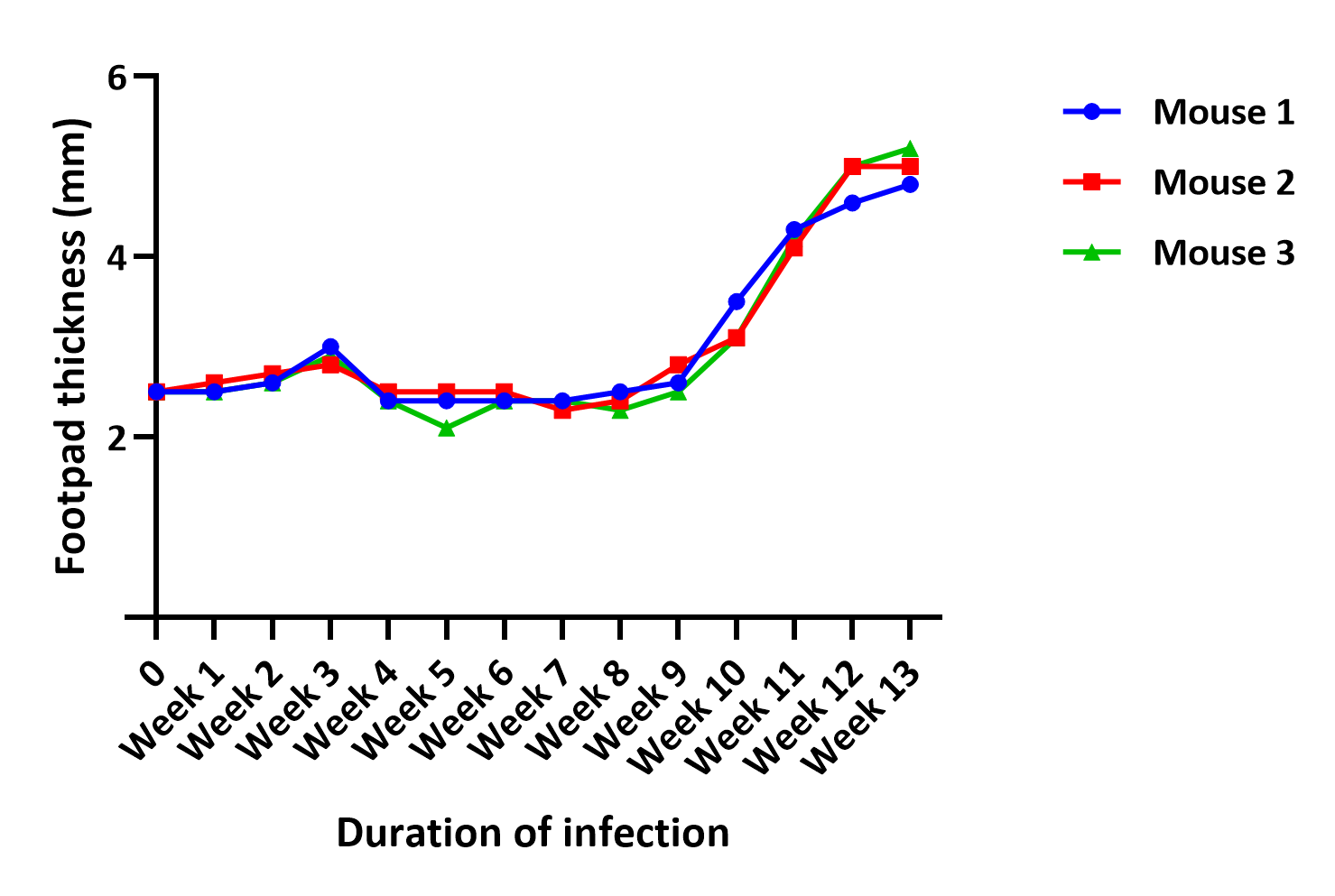

Supplement: S1 Fig — (TIF) [file pntd.0008357.s001.tif]

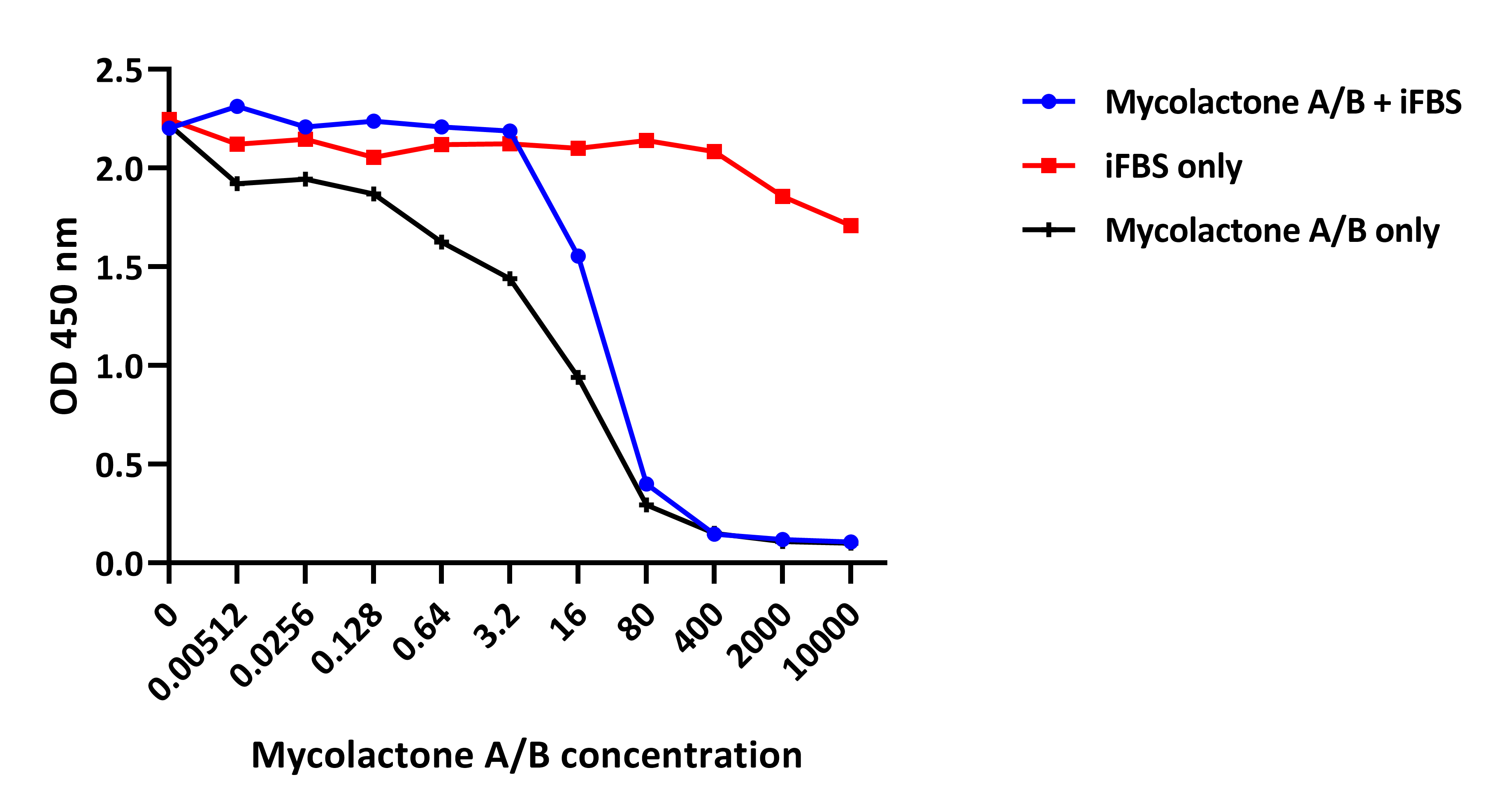

Supplement: S2 Fig — Dilution series of synthetic mycolactone A/B were done in LW buffer alone or LW buffer containing 50% fetal bovine serum (FBS) were allowed to react with mAb JD5.1 bound to MaxiSorp plates for 2 hours; plain FBS was included as negative control. Subsequently, MG-161 (40 ng/ml) was added for 45 min, and bound reporter was detected with HRP-conjugated streptavidin and TMB. (TIF) [file pntd.0008357.s002.tif]
